# Sorghum Allelopathy: Alternative Weed Management Strategy and Its Impact on Mung Bean Productivity and Soil Rhizosphere Properties

**DOI:** 10.3390/life12091359

**Published:** 2022-08-31

**Authors:** Raza Ullah, Zubair Aslam, Houneida Attia, Khawar Sultan, Khalid H. Alamer, Muhammad Zeeshan Mansha, Ashwaq T. Althobaiti, Najla Amin T. Al Kashgry, Badreyah Algethami, Qamar uz Zaman

**Affiliations:** 1 Department of Environmental Sciences, University of Okara, Punjab 56300, Pakistan; 2 Department of Agronomy, University of Agriculture Faisalabad, Punjab 38040, Pakistan; 3 Department of Biology, College of Science, Taif University, P.O. Box 11099, Taif 21944, Saudi Arabia; 4 Department of Environmental Sciences, The University of Lahore-Lahore, Punjab 54590, Pakistan; 5 Biological Sciences Department, Faculty of Science and Arts, King Abdulaziz University, P.O. Box 80200, Rabigh 21911, Saudi Arabia; 6 College of Agriculture, Bahauddin Zakariya University, Bahadur Sub Campus, Layyah, Punjab 31200, Pakistan

**Keywords:** crop residues, profitability, soil fertility, soil biology, allelopathy

## Abstract

**Simple Summary:**

Plants are subjected to a variety of biotic and abiotic stresses, which affect the rhizospheric attributes and limit agricultural crop productivity. To meet the food and energy demands of the future, several diverse approaches are used for achieving more stress-tolerant and climate-flexible crops for sustainable yields. Several organic and inorganic amendments are used to ameliorate these stresses. Crop-mediated modification (crop residues and allelopathic extracts) has great effects on weed management, improving rhizospheric attributes, and ultimately producing the best quality yield. Sorghum crop residues and their allelopathic extract can be used as a nutrient resource to enhance soil and crop productivity through their application. Sorghum-mediated crop modification will support the soil health and environmental sustainability, providing insight into the improvement of crop productivity. This study will help policymakers in modelling and enhancing sustainable crop production.

**Abstract:**

The reduction of herbicide use and herbicide-resistant weeds through allelopathy can be a sustainable strategy to combat the concerns of environmental degradation. Allelopathic crop residues carry great potential both as weed suppressers and soil quality enhancers. The influence of sorghum crop residues and water extracts on the weed population, soil enzyme activities, the microbial community, and mung bean crop productivity was investigated in a two-year experiment at the Student Research Farm, University of Agriculture Faisalabad. The experimental treatments comprised two levels of sorghum water extract (10 and 20 L ha^−1^) and two residue application rates (4 and 6 t ha^−1^), and no sorghum water extract and residues were used as the control. The results indicated that the incorporation of sorghum water extract and residue resulted in significant changes in weed dynamics and the soil quality indices. Significant reduction in weed density (62%) and in the dry weight of weeds (65%) was observed in T_5_. After the harvest, better soil quality indices in terms of the microbial population (72–90%) and microbial activity (32–50%) were observed in the rhizosphere (0–15 cm) by the same treatment. After cropping, improved soil properties in terms of available potassium, available phosphorus soil organic matter, and total nitrogen were higher after the treatment of residue was incorporated, i.e., 52–65%, 29–45%, 62–84%, and 59–91%, respectively. In the case of soil enzymes, alkaline phosphatase and dehydrogenase levels in the soil were 35–41% and 52–77% higher, respectively. However, residue incorporation at 6 t ha^−1^ had the greatest effect in improving the soil quality indices, mung bean productivity, and reduction of weed density. In conclusion, the incorporation of 6 t ha^−1^ sorghum residues may be opted to improve soil quality indices, suppress weeds, harvest a better seed yield (37%), and achieve higher profitability (306 $ ha^−1^) by weed suppression, yield, and rhizospheric properties of spring-planted mung beans. This strategy can provide a probable substitute for instigating sustainable weed control and significant improvement of soil properties in the mung bean crop, which can be a part of eco-friendly and sustainable agriculture.

## 1. Introduction

The human population will be 8.6 billion by 2030, as estimated by the United Nations, and is projected to be 9.8 billion by 2050 [1]. To feed this many humans, there must be an increase of 40% to 70% in food production [2] to feed the growing number of people by the year 2050. Food production increased by about 146% between 1961 and 2000, while the agricultural land for crops increased by only 8% [3]. This milestone was reached by an intensive use of topsoil nutrients and agrochemicals, rendering the soil exhausted and polluted [4]. Geographically, a large part of Pakistan is located in a dry-land environment where 80% of the land is classified as arid to semi-arid and only 8% is humid [5]. More than 60% of Pakistan’s population depends on dry land to support their life and income, mainly through agriculture and pastoral activities [6]. Sustainable land management and crop production systems are necessary for developing countries to achieve stable production in the food supply system [5,6].

Weeds are another major concern in agriculture, and a large number of herbicides have been manufactured and used in soil [7]. The rhizosphere contains millions of weed seeds that grow when they get suitable growing conditions, otherwise remaining dormant [7]. Of the total pesticides manufactured around the world, 15% are herbicides [8]. Although the use of herbicides in Pakistan is higher because of the availability of labor for manual weeding, that is changing fast due to the introduction of mechanized systems in agriculture, and the tendency of applying chemical herbicides is on the rise with time [7].

Furthermore, the rise in the application of herbicides for managing weeds is a serious environmental risk to organisms and the planet. According to government statistics, Pakistan has seen an increase in the use of the pesticide glyphosate. In 2015, over 1100 tons of glyphosate were imported from other countries. In 2020, this number increased to 2000 tons, with importers including both domestic and foreign pesticide manufacturers [9]. Excessive herbicide use may result in a shift with the emergence of herbicide-resistant weeds and related health problems, which have changed the research interest to find alternative tools for managing weeds [10]. Finding more sustainable alternative options for weed control that will decrease the dependence on traditional farming practices, including synthetic herbicides, need time [11].

Allelopathy is the best substitute compared to synthetic herbicides, as allelochemicals have no residual toxic effects; however, many allelochemicals have limited efficacy and specificity [12,13]. Allelopathic crops carry great potential for the development of cultivars that are weed-suppressive. The application of residue of allelopathic crops suppresses weeds and improves soil health and crop production [14]. Crop residue is not only an excellent source of nutrients, but a significant source of organic material applied to soils as it improves soil health by increasing nutrient and water-holding potential [13]. Many crop plants including sorghum have been widely used for allelopathy. Besides these allelopathic properties, sorghum ranks among the top three important grains, as its industrial demand is increasing particularly in the food, beverage, and livestock feed industries [14]. Recently, sorghum has also gained interest as a new-generation bioenergy crop because of its multiple uses and wide adaptability to varied agroclimatic conditions [15]. Crops such as sunflower, sorghum, wheat, rice, rye, barley, maize, cucurbits, and alfalfa all show strong allelopathic potential. Among them, sorghum is the most investigated crop concerning its allelopathic potential [16]. The application of crushed sorghum mulch significantly decreased the total weed dry weight (26–56%) with a yield increase of 6–17% in wheat crops [17,18]. In cotton and maize, the use of sorghum surface mulch substantially decreased the weed population with a significant crop yield increase [11,16,19]. Species-specific compounds found in root exudates have important ecological consequences on soil health (macro- and microbiota) and plant health. Symbiotic relationships are supported and soil qualities, such as chemical and physical properties, are altered by the exudation of diverse substances [20]. The concentrations and classifications of these metabolites have a direct effect on ion absorption. For example, N and K absorption is boosted by a modest concentration of dibutyl phthalate and diphenylamine [16,20].

In the rhizospheric biome, there are millions of bacteria that have positive interactions with the plants and promote their growth and survival, while only a few are found to be pathogenic to plants [21]. The beneficial bacteria stimulate plant growth, make nutrients available to plants, suppress the growth of pathogens, and improve the soil structure, thus playing an essential role in sustainable crop production [3,7,11]. These beneficial microbes in the rhizosphere decompose the added residues which results in the improvement of soil health such as soil organic C sequestration, microbial biomass C, activity of soil biota [22], increase in soil organic matter, reduction in the fertilizer cost, and weed control, which ultimately results in better production [23].

A wide range of factors, including soil health, production costs, net revenue per acre, crop yields, gross income per acre, individual farm income, and many more can be improved through sustainable agriculture [16]. Organic and sustainable farming practices move us one acre closer to sustainability, or at the very least one acre less likely to cause harm [24]. Some studies have documented weed control by using sorghum water extract and sequential plantation of sorghum [7,11,16,24]. However, not much is known about the possible changes of such uses of allelopathic interventions on the soil–plant environment and microbial diversity. In this study, we hypothesized that sorghum water extracts and residues may suppress weeds while improving soil health and mung bean productivity, which is the only viable option available for meeting the growing demand for food in developing countries. The precise objective of the experiment was to find out the impact of sorghum water extracts and the residues upon soil enzymatic and chemical activities, weed dynamics, the population of microbes, and mung bean productivity by the modulation of physiological parameters.

## 2. Materials and Methods

### 2.1. Experimental Site, Climate and Soil Sampling

The experiment in this study was conducted at the Student Research Farm, Department of Agronomy (University of Agriculture Faisalabad), Pakistan (Latitude: 31°26′ N and Longitude: 73°06′ E; Altitude: 184.4 mASL). As per the classification system, the soil belongs to the Lyallpur series. According to the US Department of Agriculture classification system, the soil type is arid sol-fine-silty, hyperthermic Ustalfic, mixed, and Haplargid. According to the Food and Agriculture Organization classification system, it is classified as Haplic Yermosols soil type.

The soil samples from mung bean plants’ rhizosphere were sampled 20 days after planting and the following harvest. Before the experiment began, a composite sample of ten soil samples (0–15 cm) was taken from the experimental site. A second composite sample was then taken from the same field after the crop had been harvested to determine the enzymatic characteristics and microbial counts of the soil samples. The soil samples were subjected to air drying, grinding, and sieving (using a 2 mm sieve), and all parameters except for microbial culture and dehydrogenase activity were examined. Soil samples were kept at 4 °C for both dehydrogenase and microbiological analysis. Soil properties, nutrient dynamics, soil enzyme activities, and microbial populations of the experimental soil were measured before sowing by following standard protocols as depicted in Table 1.

The field data related to weather parameters for the whole period of crop growth and management were taken from the Meteorological Observatory, Department of Agronomy, University of Agriculture, Faisalabad, Pakistan and are given in Table 2.

### 2.2. Experimental Treatments and Design

The field experiment in this study was designed with the treatments: control (plots with no crop residues or extract application), sorghum water extract at 10 L ha^−1^, sorghum water extract at 20 L ha^−1^, sorghum residues at 4 t ha^−1,^ and sorghum residues at 6 t ha^−1^. The field experiment design involved a randomized complete block design (RCBD) with three replications. The size (area) of each plot for each treatment was measured to be 15 m^2^ (3.0 m × 5.0 m).

### 2.3. Crop Management

The experimental site was ploughed twice using a tractor-drawn cultivator and then planked. Flat wooden planks were utilized for breaking up clods, and a laser leveler was used for leveling soil. Wheat was the fore-crop for mung beans. The seed of mung bean cultivar NM-92 was collected from the National Institute of Agriculture and Biology (NIAB), Faisalabad. It was planted on 15 March 2014 and repeated on 20 March 2015. A recommended rate of mung bean seeds (25 kg ha^−1^) was used to maintain the plant population in 30 cm apart rows using a hand drill. Urea, diammonium phosphate, and sulphate of potash were applied at the rate of 3 kg N, 58 kg P_2_O_5_, and 63 kg K_2_O ha^−1^ for the nutrient requirement. The recommended dose of P and K and 1/3rd of N was applied at the time of sowing, and the remaining N was applied with the first and second irrigation using the top-dressing method. After ten days of sowing, the first irrigation was applied (7.5 cm), while subsequent irrigation was applied upon crop requirement. To control the termites and pod borers, insecticides Fipronil and Emamectin Benzoate were applied at the rate of 1.73% *w/w* ha^−1^ and 5.03% *w/w* ha^−1^, respectively. The crop harvesting was done on the 10th and 15th of July during both years.

### 2.4. Sorghum Crop Water Extracts Preparation

Sorghum plant residue samples were obtained from the Student Research Farm (University of Agriculture Faisalabad). The plant samples were harvested at maturity, shade dried, and cut into pieces (<3 cm in size) by using the electric fodder cutter. These shredded pieces of sorghum residues were then placed in distilled water for 24 h with a ratio of 1:10 (*w*/*v*%) and the filtrate liquid obtained from it was used as fresh [25]. This sorghum water extract was used as 10 & 20 L ha^−1^ by spraying (300 L ha^−1^) with the help of a T-jet nozzle using a knapsack sprayer 5 days after the sowing (3–5 leaf stage) of the mung bean plants. A total volume of sprayed liquid was determined by the calibration method.

### 2.5. Sorghum Crop Residues Preparation

Samples of sorghum plant residues were also collected from Student Research Farm (University of Agriculture Faisalabad). Plants were harvested at maturity, shade dried, and then cut into tiny pieces in sizes less than 3 cm with the help of a machine (electric fodder). These dried pieces of the crop were then applied to the soil before the sowing as per treatments of 4 and 6 t ha^−1^ in the experiment.

### 2.6. Data Analysis

For the measurement of weeds-related, soil-related and yield-related attributes, the following protocols were followed.

#### 2.6.1. Soil Attributes, Microbial Population, and Soil Enzymatic Activities

Soil electrical conductivity (EC) and the pH of the soil were determined by following the protocols of Ryan et al. [26]. Water/soil suspension was utilized at a ratio of 2:1 to measure soil EC and pH. The value of EC and pH was measured using a Jenway Model 4510 digital conductivity meter and a Kent Eil 7015 pH meter. The protocols of Blake and Hartge [27] and Vomocil [28] were followed to determine the total porosity of soil (TP) and soil bulk density (BD), respectively. Similarly, methods developed by Bremner and Mulvaney [29], Walkley and Black [30], Olsen and Sommers [31], and Helmke and Sparks [32] were used for the calculation of total nitrogen (TN), available potassium (K), available phosphorus (P), and SOM (soil organic matter). The microbial populations of soil samples were measured by the method of spiral plating serial dilutions of each sample on agar plates [33]. A total number of culturable bacteria populations was measured on R2A (half-strength) agar plates following the methods described by Janssen et al. [34] and Wu et al. [35]. The culturable fungi in samples were determined using the plating method in Rose Bengal media of potato dextrose agar [36] and the colony counts were carried out after enough time (48 h) for culturing. The microbial activity as indicated by CO_2_ evolution was measured by acid-base titration procedure and reported as mg CO_2_-C kg^−1^ d^−1^. Soil dehydrogenase enzymatic activity was measured by the procedures described by Min et al. [37] and was reported as µg TPF g^−1^ 12 h^−1^. Alkaline phosphatase activity was determined spectrophotometrically by following the method described by Tabatabai and Bremner, [38] and was reported as µg p-nitrophenol g^−1^ h^−1^ in this study.

#### 2.6.2. Weeds Dynamics

Weed dynamics such as the total number of weeds (0.25 m^−2^), fresh weight (g 0.25 m^−2^), and dry weight (g 0.25 m^−2^) were observed and recorded from each plot for 30 days after sowing by randomly selecting two quadrates (50 cm × 50 cm). First, weeds were counted one by one and then cut at levels above the ground surface. For determining the dry weight, weeds were dried under the sunlight for 48 h and then dried in an electric oven for 72 h, maintaining temperatures at 70 °C. The dry weight of samples was recorded by using an electric balance after attaining the constant weight.

#### 2.6.3. Yield Attributes

The yield components such as the number of pods per plant, number of seeds per pod, and 1000-seed weight were observed and recorded by the methods described by Rab et al. [39]. Mung bean crop samples were harvested at maturity and threshed manually for the separation of seeds from straw. The seed yield of each experimental unit was recorded and expressed as kg ha^−1^ in the experiment.

#### 2.6.4. Statistical Analysis

Statistical analysis of data (both years) was carried out using Statistix 8.1. For the comparison of treatments, the LSD (least significance difference) test at 5% probability was applied.

## 3. Results

### 3.1. Weeds Dynamics

Horse purslane (*Trianthema portulacastrum*) and purple nutsedge (*Cyperus rotundus*) both were dominant in each experimental unit during both years of study. This study indicated that the density and dry weight of horse purslane significantly differed with various allelopathic weed management strategies. Total weed density and dry weight also significantly differed with various allelopathic weed management strategies. However, the dry weight of purple nutsedge was non-significant among various allelopathic weed management strategies (Table 3). The effect of time was significant for all weed parameters except the dry weight of purple nutsedge (Table 3). The interaction of allelopathic weed management strategies and the year was significant for total weed density but non-significant for the dry weight of the weeds, as well as for density and dry weight of the horse purslane and purple nutsedge (Table 3).

The lowest horse purslane (13) and purple nutsedge (3) densities were recorded with sorghum residues at 6 t ha^−1^ compared to the control (40 and 10, respectively). The lowest values were observed in the control (Table 3). Total weed density and dry weight decreased over time and the minimum values were observed during the 2nd year (Table 2). In the case of horse purslane, dry weight (14 g/0.25 m^−2^) was observed with sorghum residues at 6 t ha^−1^, followed by sorghum residues at 4 t ha^−1^ (Table 3). The maximum value of dry weight (48 g/0.25 m^−2^) was observed in the control (Table 3). In the case of total weed density, the interactive effect of allelopathic weed management strategies and the year showed a statistically significant effect. The minimum total weed density (18.42) was recorded with 6 t ha^−1^ sorghum residues during the 2nd year, as compared to the control (56.55). The lowest total weed dry weight (56.23 and 19.97 g/0.25 m^−2^, respectively) was observed with sorghum residues at 6 tons ha^−1^ and the maximum total weed density (56.85) was recorded in the control (Table 3).

### 3.2. Yield and Yield Parameters

Yield and yield parameters differed significantly among the various allelopathic weed management strategies (Table 4). Likewise, the year effect was significant for the weight of 1000 seeds, biological yield, harvest index, and yield, but non-significant for No. of pods per plant and No. of seeds per pod (Table 4). The interaction of allelopathic weed management strategies and the year was significant only for yield (Table 4). However, the interaction was non-significant for No. of pods per plant, No. of seed per pod, the weight of 1000 seeds, biological yield, and harvest index (Table 4). The results indicated that the maximum values of No. of pods per plant (24.6), No. of seed per pod (9.9), weight of 1000 seeds (55.33 g), biological yield (4106 kg ha^−1^), harvest index (26.01%), and yield (1019.3 kg ha^−1^) were recorded with sorghum residues at 6 tons ha^−1^. The minimum values of No. of pods per plant (14.6), No. of seeds per pod (5.9), weight of 1000 seeds (50.25 g), biological yield (3206 kg ha^−1^), harvest index (22.74%), and yield (744.3 kg ha^−1^) were observed in the control (Table 4). A linear increase in the No. of pods per plant, No. of seeds per pod, weight of 1000 seeds, biological yield, harvest index, and yield was observed over time and all the above observations had a significant increase in values during the 2nd year of the study (Table 4). In the present study, all treatments gave higher net returns as compared with the control during both the years of study. Among all treatments, sorghum residue at 6 tons ha^−1^ gave maximum economical returns during both years, while a minimum net benefit was obtained from the control (Table 5).

### 3.3. Rhizosphere Soil Microbial Population and Enzymes Activity

Microbiological and biochemical indicators are also used as full indicators of soil health. They are more susceptible than physical and chemical attributes to changes imposed on the environment. Microbiological indicators such as the population of bacteria, fungi, and microbial activity at 20 days after sowing and harvesting differed significantly among various allelopathic weed management strategies (Table 6). The year effect was also significant for all the above parameters (Table 6). The interactive effect of allelopathic weed management strategies and the year was significant for the population of fungi but non-significant for the population of bacteria at 20 days after sowing and at harvesting (Table 6). Biochemical indicators like soil enzymes (alkaline phosphatase and dehydrogenase) differed significantly among various allelopathic weed management strategies at harvest (Table 6).

Interaction (allelopathic weed management strategies × year) was significant for the fungal population. The highest fungal population (25 cfu/g × 10^4^ and 20 cfu/g × 10^4^, respectively) was recorded with the application of sorghum residues at 6 t ha^−1^ at both stages, i.e., 20 days after sowing and at the harvesting of the second year of the experiment. However, the highest bacterial population (79 cfu/g × 10^4^ and 42 cfu/g × 10^4^, respectively) and microbial activity (5.52 mg CO_2_-C kg^−1^ d^−1^ and 4.58 mg CO_2_-C kg^−1^ d^−1^, respectively) were recorded with the application of sorghum residues at 6 t ha^−1^ at both stages, i.e., 20 days after sowing and at harvesting. The lowest microbial activity (3.70 mg CO_2_-C kg^−1^ d^−1^) and the lowest populations of both bacteria (22 cfu/g × 10^5^) and fungi (6 cfu/g × 10^4^) were observed in the control (Table 6). A linear increase in the bacterial population was observed over time at 20 days after sowing and at harvesting, and the highest bacterial population was observed during the second year (Table 6). In the case of soil enzymes, the interactive effect of the allelopathic weed management strategies and the year resulted in a significant effect on the activity of both enzymes alkaline phosphatase and dehydrogenase. The highest value (196.22 μg NP g^−1^ soil h^−1^ and 44.00 μg TPFg^−1^ soil h^−1^, respectively) was observed with the application of sorghum residues at 6 t ha^−1^ during the second year, which was followed with the same treatment in the first year. The lowest value (135.50 μg NP g^−1^ soil h^−1^ and 23.33 μg TPFg^−1^ soil h^−1^, respectively) was recorded in the control (Table 6).

### 3.4. Rhizosphere Soil Properties and Nutrient Dynamics

At the end of the experiment, the physical indicators of soil health like soil porosity and bulk density significantly differed among various allelopathic weed management strategies (Table 7). The year effect was also statistically significant for the soil’s physical indicators but the interaction (allelopathic weed management strategies × year) was non-significant (Table 7). Chemical indicators of soil health such as EC (electrical conductivity), SOM (soil organic matter), N (nitrogen), available K (potassium), and P (phosphorus) significantly differed among various allelopathic weeds management strategies (Table 7). The year effect was also statistically significant for all soil chemical indicators except soil pH and available K. The interaction (allelopathic weed management strategies × year) was statistically significant for SOM, N, and available P. However, for soil pH, EC and available K interactions were non-significant (Table 7). The lowest bulk density (1.30 g cm^−3^) and the highest soil porosity (50.22%) were observed in treatments when sorghum residues at 6 tons ha^−1^ were applied, as compared to the control, while the lowest bulk density (1.23 g cm^−3^) and highest soil porosity (51.79%) were observed in second year of the experiment (Table 7). In case of SOM, N, and available P, the highest values (1.37%, 0.45 g kg^−1^, 10.31 mg kg^−1^, respectively) were observed during the second year when sorghum residues at 6 tons ha^−1^ were applied as compared to the control (0.69%, 0.21 g kg^−1^, 6.77 mg kg^−1^, respectively). Among all allelopathic weed management strategies, the statistically highest values of soil EC (1.34 dS m^−1^) and available K (200.83 mg kg^−1^) were obtained with the application of sorghum residues at 6 tons ha^−1^. The statistically lowest values for all parameters given above were observed in the control, which was statistically similar to sorghum water extracts at 10 and 20 L ha^−1^ (Table 7). A linear increase in soil EC and available K was observed over time, and these parameters (soil EC and available K) had the highest values during the second year of the experiment (Table 7). In the case of soil pH, a decreasing trend was observed. The lowest soil pH (7.28) was observed with the application of sorghum residues at 6 tons ha^−1^ and the highest soil pH (7.73) was observed in the control, which was statistically similar to the sorghum water extract at 10 and 20 L ha^−1^ (Table 7).

## 4. Discussion

Incorporating allelopathic crop residues is a green approach to manage weeds in field crops. Our results showed significant weed suppression potential with the incorporation of sorghum residues and water extract. This approach had a maximum reduction in weed density, fresh weight, and dry weight of weed species in mung bean crops (Table 3). This reduction was due to the release of phenolic compounds, including phenolic acids (Dhurrin, p-hydroxybenzaldehyde, sorgoleone, vanillic acid, p-hydroxybenzoic acid, p-hydroxybenzaldehyde, p-coumaric acid, and ferulic acid) with a wide spectrum of biological activities, including allelopathy [40,41]. In the case of field crops, sorghum had the highest allelopathic potential, which has been reported by many researchers [16,17,42]. Inhibitory activity of sorghum allelochemicals on grassy and broad-leaved weeds has been reported [17]. Cheema and Khaliq [18] investigated that 35–49% of weed density and weed biomass was reduced by using water extract of mature sorghum crop plants as compared with the control group. The sorghum residue treatments showed the highest suppression of weeds compared to sorghum water extracts treatments (Table 3); adding sorghum at 2–6 Mg ha^−1^ to the soil reduced the weed biomass by 40–50%. Crop residues may change the weed frequency and distribution, and may cause the suppression of weeds [14,43]. Zaji and Majd [44] showed that the fresh weight and dry weight of different weed biota, viz., red root pigweed (*Amaranthus retroflexus*), palmer amaranth (*Amaranthus palmeri*), black nightshade or wonder berry (*Solanum nigrum*), and curled dock (*Rumex crispus*) were decreased severely by the impact of canola crop residues. The growth suppression of dominant weed biota in this experiment might have been observed due to the physical resistance by sorghum residues’ incorporation or the release of chemicals from these residues [7]. Allelochemicals released through different parts of plants are dependent on many factors, i.e., applied crop family, size and dose of mulching, decomposition rate, moisture contents, the texture of the soil, and soil microbiota [45,46]. Weed suppression level is directly related to the dose of allelopathic products [47,48]. The higher the amount of plant material used for mulch, the greater the total amount of allelochemicals present in the mulch and released, leading to a higher concentration of allelochemicals into the soil [49,50,51]. Generally, by incorporating a higher amount of crop residues, greater weed suppression was observed. A two-year field experiment was conducted by Alsaadawi et al. [52] who stated that sorghum residue incorporation significantly reduced the weed number and produced a higher yield of broad bean than weedy check.

In our study, more than a 37% increase in mung bean yield was achieved through effective allelopathic weed management strategies (Table 4). This increase in crop yield might be due to the improvement of soil properties and reduced weed competition during the critical periods of crop growth. The effective reduction of weeds also increases the obtainability of resources such as light, moisture, nutrients, and yield gap [7,53]. Research on wheat residue application in the Mediterranean environment by Stagnari et al. [54] concluded that the conservation of soil moisture was improved, especially during the critical growth period of the test crop. The residues which are completely decomposed into the soil not only provide allelochemicals, but also participate in nutrition for crop plants. They provide nitrogen by releasing it into the rhizosphere soil of the tested crop plant. The application of sorghum residues as biological weed management helps in the mineralization of nitrogen and enhances nitrogen availability in the rhizosphere [7,14,50]. However, at later stages of crop growth, the obtainability of nitrogen was improved by mineralization, so this sustained supply of nitrogen was a nonstop source of nutrition for test crops as well as next crops. Therefore, the incorporation of sorghum residues improved soil properties, viz., moisture retention; restored physical properties; enhanced nutrient cycling and microbial activity due to the presence of phenolic compounds [55,56,57]; and suppressed weeds due to the physical hindrance by residues, reduced light penetration, and the suppressing ability of allelochemicals, which, released from these plant residues, harvested better seed yield and achieved higher profitability in spring-planted mung bean [16,45,46,58].

Our results indicated that using sorghum residues as allelopathic weed management strategies in mung bean crops improved the microbial population and enzymatic activities of the soil (Table 6). Microbial abundance and soil enzymes are biological soil activities and important indicators of soil quality [59,60,61]. The incorporation of different crop residues in the soil modified the bio-chemical attributes, i.e., soil microbial population and soil enzymatic activity [62]. Soil enzymes and microbiota play a key role in the availability of nutrients. The dehydrogenase enzyme is important for the oxidation of soil organic matter (SOM), transferring the hydrogen and electrons from substrates to acceptors. The activity of soil enzymes, viz., dehydrogenase and phosphatase, depends on the type of residues incorporated in the soil. It also depends on the moisture content and the temperature of the soil. It affects the activity of dehydrogenase by changing the oxidation-reduction status of soil [63,64]. Incorporation of crop residues, viz., tobacco and sunflower in the soil increased the activities of most of the soil enzymes, while the residues of tomato crop only increased the activity of amylase and phosphodiesterase [16,65]. In Akola, Maharashtra, Ravankar et al. [66] reported that the incubation of soil with 1% organic residues, stalks, straw, stubble, stovers, trash, and husks of various field crops showed a wide variation in the rate of decomposition, C:N ratio, and the microbial population at different intervals. Fungal, bacterial, and actinomycetes populations increased after 30 days of incubation. Bacteria were predominant over fungi and actinomycetes.

The incorporation of sorghum residues not only had a positive effect in the case of reducing the weed population and biomass, but also improved nodulation and nitrogen fixation processes, as well as the physical, chemical, and nutritional statuses of field soils. Our results indicate that increased quantities of crop residues have decreased the bulk density and increased the total porosity of the soil over time (Table 7). Soil porosity is directly related to the soil bulk density because as soil bulk density decreases, the soil porosity increases [67]. In the case of soil properties, sorghum residues as an allelopathic weed management strategy improved the SOM, N, available K, and P in the soil (Table 7). Crop residues are good sources of nutrients and are the primary source of organic material added to the soil [68]. They increase the nutrient availability and water-holding capacity of the soil [69]. Moisture retention is the main benefit of residue incorporation. It is caused by a decrease in runoff and evaporation of water from the soil [70,71]. The improvement in nutrient accumulation (especially P and K) might be attributed to the enhanced moisture retention within the soils [16,72]. Improved moisture availability due to residue incorporation also indicated that the soil’s water-holding capacity was improved and the soil moisture was available for longer times to support plant growth [73]. This increase in moisture retention properties might decrease the irrigational requirements of the crops, which should be investigated in future studies. In one study, Raut et al. [74] stated that incorporation of sunflower straw at 4 t ha^−1^ and RDF at (125% N + 100% P) in green gram recorded significantly higher soil N, K, and P content in green gram–sunflower sequence. As a result, the incorporation of sorghum residues increased the soil’s physical characteristics, microbial activity, and nutrient cycling [55,56,75,76]. The decreased chance of light penetration and the potential of allelochemicals emitted from the plant debris to limit growth also repressed weeds [46,65,77]. The spring-planted mung bean crop was more profitable and produced a higher yield of seeds as a result of all the aforementioned operations.

## 5. Conclusions

Due to their direct mode of action on the soil surface, herbicides are hazardous to both plants and soil microbes. Weeds and soil quality were significantly impacted by the sorghum crop’s allelopathy. In our study, the differential ability to suppress weeds was observed among various sorghum residue and water extract application treatments. A high suppression of weed density, fresh weight, and dry weight was observed when sorghum residues were incorporated into the soil at 6 t ha^−1^. The residues favorably affected the soil properties, viz., microbial populations, activity, and soil enzymes. The improvement in soil properties and the suppression of weeds harvested a better seed yield and achieved higher profitability in spring-planted mung bean. In short, sorghum residue implementation may provide better weed control along with enhancing the soil health and seed yield of spring-planted mung bean. Different multidisciplinary approaches that incorporate sorghum crops for strategic weed control might be potential alternatives that can also serve as lead compounds for herbicide discovery programs. Future studies should also focus on the interactions of micronutrients in the soil environment under multiple allelopathic weed management techniques in the field. Additionally, there are still relevant issues to look into regarding nitrogen and weed management using different allelopathic strategies and observing the allelopathic effect between the crop residue and the crop that is applied.

## Figures and Tables

**Table 1:** Soil properties, nutrient dynamics, soil enzyme activities, and microbial populations of the experimental soil before sowing.

Soil Indices	Experiment Year 1	Experiment Year 2
pH	7.85	7.79
Electrical Conductivity (dS m^−1^)	1.11	1.19
Total Soil Organic Matter (%)	0.53	0.61
Available Phosphorous (mg kg^−1^)	6.74	6.95
Available Potassium (mg kg^−1^)	123.00	131.00
Total Soil Nitrogen (g kg^−1^)	0.24	0.29
Bacteria (cfu/g × 10^5^)	35.00	45.00
Fungi (cfu/g × 10^4^)	5.00	8.00
Microbial Activity (mg CO_2_-C kg^−1^ d^−1^)	3.05	3.14
Alkaline Phosphatase Activity (μg NP g^−1^ soil h^−1^)	135.00	143.00
Dehydrogenase Activity (μg TPFg^−1^ soil h^−1^)	21.00	25.00

**Table 2:** Weather indices of an experimental site for the study period.

Months	Weather Indices	Experiment Year 1	Experiment Year 2
March	Maximum Temperature (°C)	25	25
	Minimum Temperature (°C)	14	14
	Rain Fall (mm)	42	68
	Relative Humidity (%)	60	64
April	Maximum Temperature (°C)	32	33
	Minimum Temperature (°C)	19	21
	Rain Fall (mm)	28	33
	Relative Humidity (%)	52	44
May	Maximum Temperature (°C)	37	39
	Minimum Temperature (°C)	24	25
	Rain Fall (mm)	41	17
	Relative Humidity (%)	33	28
June	Maximum Temperature (°C)	41	38
	Minimum Temperature (°C)	28	26
	Rain Fall (mm)	7	12
	Relative Humidity (%)	34	39
July	Maximum Temperature (°C)	37	35
	Minimum Temperature (°C)	28	27
	Rain Fall (mm)	58	128
	Relative Humidity (%)	54	61

**Table 3:** Effect of sorghum water extracts and residues on weed dynamics in mung bean crops.

Treatments	Year 1	Year 2	Mean ^(a)^ (T)	Year 1	Year 2	Mean (T)	Year 1	Year 2	Mean (T)
	Horse Purslane Density (0.25 m^−2^)			Horse Purslane Fresh Weight (g/0.25 m^2^)			Horse Purslane Dry Weight (g/0.25 m^2^)		
T_1_	41	40	40 A	156	147	152 A	49	47	48 A
T_2_	41	32	36 B	135	126	130 B	40	43	41 B
T_3_	38	27	32 C	118	99	108 C	37	32	34 C
T_4_	22	16	19 D	82	65	73 D	26	21	23 D
T_5_	13	12	13 E	48	43	46 E	15	13	14 E
Mean ^(b)^ (Y)	31 A	26 B		104 A	100 B		33 A	31 B	
*LSD* (*p* ≤ 0.05)	*T* = *3.76*; *Y* = *2.38*			*T* = *14.19*; *Y* = *3.15*			*T* = *4.51*; *Y* = *1.85*		
	**Purple Nutsedge Density** **(0.25 m^−2^)**			**Purple Nutsedge Fresh Weight (g/0.25 m^2^)**			**Purple Nutsedge Dry Weight (g/0.25 m^2^)**		
T_1_	10	10	10 A	13	13	13	4	4	4
T_2_	9	7	8 B	10	6	8	3	2	3
T_3_	7	7	7 C	6	6	6	2	2	2
T_4_	6	5	5 D	3	3	3	1	1	1
T_5_	3	2	3 E	3	3	3	1	1	1
Mean (Y)	7 A	6 B		7	6		2	2	
*LSD* (*p* ≤ 0.05)	*T* = *1.09*; *Y* = *0.68*			*NS*			*NS*		
	**Total Weed Density** **(0.25 m^−2^)**			**Total Weed Fresh Weight (g/0.25 m^2^)**			**Total Weed Dry Weight (g/0.25 m^2^)**		
T_1_	57.15 a	56.55 a	56.85 A	175.18	165.94	170.56 A	58.05	55.12	56.58 A
T_2_	52.93 ab	49.61 bc	51.27 B	147.90	141.61	144.76 B	49.39	47.39	48.39 B
T_3_	45.77 c	36.55 d	41.16 C	130.28	112.08	121.18 C	43.79	38.02	40.91 C
T_4_	35.98 d	25.78 e	30.88 D	88.16	78.70	83.93 D	31.37	26.15	28.76 D
T_5_	24.35 e	18.42 f	21.39 E	59.78	52.68	56.23 E	20.78	19.16	19.97 E
Mean (Y)	43.12 A	37.50 B		120.26 A	110.20 B		39.69 A	38.15 B	
*LSD* (*p* ≤ 0.05)	*T* = *3.81*; *Y* = *2.41*; *T* × *Y* = *5.39*			*T* = *14.44*; *Y* = *8.13*			*T* = *4.59*; *Y* = *0.55*		

Figures of interaction and main effects sharing the same case letter do not differ significantly (*p* ≤ 0.05) by the least significant difference test; likewise, the figures of main effects and interaction without lettering do not differ significantly (*p* ≤ 0.05) by the least significant difference test; T_1_ = Control (plots with no crop residues or extract application); T_2_ = Sorghum water extract at 10 L ha^−1^; T_3_ = Sorghum water extract at 20 L ha^−1^; T_4_ = Sorghum residues at 4 t ha^−1^; T_5_ = Sorghum residues @ 6 t ha^−1^; ^(a)^ T = treatments; ^(b)^ Y = year.

**Table 4:** Effect of sorghum water extracts and residues on yield and yield components of mung bean plants.

Treatments	Year 1	Year 2	Mean ^(a)^ (T)	Year 1	Year 2	Mean (T)	Year 1	Year 2	Mean (T)
	Final Emergence Count per Plot			Plant Height at Maturity (cm)			Number of Nodules per Plant		
T_1_	557	558	558	40.7	41.2	40.9 C	5	5	5 C
T_2_	558	560	559	42.5	42.7	42.6 C	7	8	8 B
T_3_	560	562	561	42.6	43.7	43.1 C	7	8	8 B
T_4_	561	562	562	45.5	46.6	46.0 B	9	9	9 AB
T_5_	563	565	564	47.7	49.0	48.3 A	10	11	11 A
Mean ^(b)^ (Y)	560	561		44.0	44.4		8	8	
*LSD* (*p ≤* 0.05)	*NS*			*T* = *2.1*			*T* = *1.79*		
	**No. of Pods per Plant**			**No. of Seeds per Pod**			**Weight of 1000-Seeds** (**g**)		
T_1_	13.76	15.33	14.55 D	5.43	6.37	5.90 E	49.95	50.54	50.25 E
T_2_	17.00	19.09	18.05 C	6.55	7.80	7.17 D	52.58	54.03	53.31 D
T_3_	19.45	21.19	20.32 BC	6.95	8.02	7.48 C	53.25	54.90	54.08 C
T_4_	20.03	23.99	22.32 AB	7.07	9.01	8.04 B	53.76	55.66	54.71 B
T_5_	23.55	25.72	24.63 A	9.24	10.61	9.92 A	54.49	56.16	55.33 A
Mean (Y)	17.96	21.86		7.05	8.36		52.81 B	54.26 A	
*LSD* (*p ≤* 0.05)	*T* = *2.62*			*T* = *0.25*			*T* = *0.59*; *Y* = *1.45*		
	**Biological Yield (kg ha^−1^)**			**Harvest Index (%)**			**Yield (kg ha^−1^)**		
T_1_	3196	3216	3206 E	22.62	22.85	22.74 C	741.9 e	746.7 e	744.3 E
T_2_	3351	3410	3380 D	22.48	23.07	22.78 C	789.2 d	811.5 d	800.4 D
T_3_	3525	3587	3556 C	23.95	24.15	24.05 B	844.1 c	867.6 c	855.8 C
T_4_	3660	3670	3665 B	24.26	24.74	24.50 B	931.2 b	934.2 b	932.7 B
T_5_	3970	4242	4106 A	25.67	26.35	26.01 A	1009.1 a	1029.4 a	1019.3 A
Mean (Y)	3540 B	3625 A		23.80 B	24.23 A		863.70 B	877.29 A	
*LSD* (*p ≤* 0.05)	*T* = *105.07*; *Y* = *75.92*			*T* = *0.39*; *Y* = *0.41*			*T* = *21.97*; *Y* = *11.45*; *T* × *Y* = *31.07*		

Figures of interaction and main effects sharing the same case letter do not differ significantly (*p* ≤ 0.05) by the least significant difference test; likewise, the figures of main effects and interaction without lettering do not differ significantly (*p* ≤ 0.05) by the least significant difference test; T_1_ = Control (plots with no crop residues or extract application); T_2_ = Sorghum water extract at 10 L ha^−1^; T_3_ = Sorghum water extract at 20 L ha^−1^; T_4_ = Sorghum residues at 4 t ha^−1^; T_5_ = Sorghum residues at 6 t ha^−1^; ^(a)^ T = treatments; ^(b)^ Y = year.

**Table 5:** Economics of mung bean crops grown in various allelopathic weed management strategies.

Treatments	Yield (kg ha^−1^)	Adjusted Yield (kg ha^−1^)	Gross Income ^(d)^ $ ha^−1^	Total Cost $ ha^−1^	Net Benefits $ ha^−1^	Benefit–Cost Ratio
^(a)^ Control	744	670	750	615	135	0.22
^(b)^ SWE at 10 L ha^−1^	800	720	806	628	179	0.29
SWE at 20 L ha^−1^	856	770	863	633	230	0.36
^(c)^ SR at 4 tons ha^−1^	933	840	940	688	252	0.37
SR at 6 tons ha^−1^	1019	917	1027	721	306	0.42
Remarks	$44.67/40 kg					

^(a)^ Control = (plots with no crop residues or extract application); ^(b)^ SWE = sorghum water extract; ^(c)^ SR = sorghum residues; ^(d)^ $ = US dollar.

**Table 6:** Effect of sorghum water extracts and residues on microbial population, microbial activity, and soil enzymatic activity in the rhizosphere of mung bean crops.

Treatments	Year 1	Year 2	Mean ^(a)^ (T)	Year 1	Year 2	Mean (T)	Year 1	Year 2	Mean (T)	Year 1	Year 2	Mean (T)
	Bacteria (cfu/g × 10^5^) 20 ^(c)^ DAS			Fungi (cfu/g × 10^4^) 20 DAS			Microbial Activity (mg CO_2_-C kg^−1^ d^−1^) 20 DAS			Alkaline Phosphatase (μg NP g^−1^ Soil h^−1^)		
T_1_	43	44	43 D	7 d	8 d	8 C	3.63	3.77	3.70 C	135.47 e	135.50 e	135.48 C
T_2_	46	48	47 CD	8 d	8 d	8 C	3.65	3.78	3.71 C	135.48 e	135.55 e	135.52 C
T_3_	48	49	48 C	8 d	9 d	9 C	3.72	3.81	3.77 C	135.78 e	135.85 e	135.82 C
T_4_	64	74	69 B	15 c	20 b	18 B	4.88	5.05	4.97 B	167.26 d	173.46 c	170.36 B
T_5_	74	83	79 A	21 b	25 a	23 A	5.45	5.58	5.52 A	185.24 b	196.22 a	190.73 A
Mean ^(b)^ (Y)	55 B	60 A		12 B	14 A		4.26 B	4.40 A		151.85 B	155.32 A	
*LSD* (*p ≤ 0.05*)	*T* = *4.72*; *Y* = *2.98*			*T* = *1.55*; *Y* = *0.98*; *T* × *Y* = *2.20*			*T* = *0.20*; *Y* = *0.13*			*T* = *3.85*; *Y* = *2.43*; *T* × *Y* = *5.44*		
	**Bacteria (cfu/g × 10^5^) ^(d)^ AH**			**Fungi (cfu/g × 10^4^) AH**			**Microbial Activity** **(mg CO_2_-C kg^−1^ d^−1^) AH**			**Dehydrogenase (μg TPFg^−1^ Soil h^−1^)**		
T_1_	21	22	22 C	5 f	6 f	6 D	2.99	3.13	3.06 C	22.68 d	23.33 d	23.00 C
T_2_	23	24	24 C	6 f	7 f	7 CD	3.05	3.15	3.10 C	23.09 d	23.59 d	23.34 C
T_3_	24	25	25 C	6 f	9 e	8 C	3.08	3.17	3.13 C	23.33 d	24.19 d	23.76 C
T_4_	35	40	38 B	11 d	17 b	14 B	3.99	4.11	4.05 B	32.00 c	38.00 b	35.00 B
T_5_	40	44	42 A	14 c	20 a	17 A	4.50	4.65	4.58 A	37.33 b	44.00 a	40.67 A
Mean (Y)	29 B	31 A		9 B	12 A		3.52 B	3.64 A		27.69 B	30.62 A	
*LSD* (*p ≤ 0.05*)	*T* = *2.21*; *Y* = *1.40*			*T* = *1.21*; *Y* = *0.76*; *T* × *Y* = *1.71*			*T* = *0.28*; *Y* = *0.10*			*T* = *2.18*; *Y* = *1.37*; *T* × *Y* = *3.08*		

Figures of interaction and main effects sharing the same case letter do not differ significantly (*p* ≤ 0.05) by the least significant difference test; likewise, the figures of main effects and interaction without lettering do not differ significantly (*p* ≤ 0.05) by the least significant difference test; T_1_ = Control (plots with no crop residues or extract application); T_2_ = Sorghum water extract at 10 L ha^−1^; T_3_ = Sorghum water extract at 20 L ha^−1^; T_4_ = Sorghum residues at 4 t ha^−1^; T_5_ = Sorghum residues at 6 t ha^−1^; ^(a)^ T = treatments; ^(b)^ Y = year; ^(c)^ DAS = days after sowing; ^(d)^ AH = after harvesting.

**Table 7:** Effect of sorghum water extracts and residues on soil properties and nutrient dynamics in the rhizosphere of mung bean crops at harvest.

Treatments	2014	2015	Mean ^(^^a)^ (T)	2014	2015	Mean (T)	2014	2015	Mean (T)	2014	2015	Mean (T)
	Soil Bulk Density (g cm^−3^)			Total Soil Porosity (%)			Soil pH			Soil EC (dS m^−1^)		
T_1_	1.48	1.46	1.47 A	42.82	43.73	43.27 C	7.75	7.72	7.73 A	1.07	1.11	1.09 C
T_2_	1.47	1.46	1.46 A	43.53	44.10	43.82 C	7.75	7.70	7.73 A	1.11	1.14	1.12 C
T_3_	1.47	1.46	1.46 A	43.80	44.11	43.96 C	7.74	7.69	7.72 A	1.12	1.14	1.13 C
T_4_	1.41	1.29	1.35 B	48.50	50.39	49.44 B	7.44	7.41	7.43 B	1.23	1.27	1.25 B
T_5_	1.38	1.23	1.30 C	48.66	51.79	50.22 A	7.38	7.18	7.28 C	1.32	1.36	1.34 A
Mean ^(^^b)^ (Y)	1.44 A	1.38 B		45.46 B	46.83 A		7.61 A	7.54 B		1.17 B	1.20 A	
*LSD* (*p ≤ 0.05*)	*T* = *0.04*; *Y* = *0.06*			*T* = *0.75*; *Y* = *0.92*			*T* = *0.12*; *Y* = *0.04*			*T* = *0.07*; *Y* = *0.02*		
	**Total Soil Organic Matter (%)**			**Total Soil Nitrogen (g kg^−1^)**			**Available Potassium (mg kg^−1^)**			**Available Phosphorous (mg kg^−1^)**		
T_1_	0.67 d	0.69 d	0.68 C	0.22 d	0.21 d	0.22 C	121.45	121.93	121.69 C	6.74 d	6.77 d	6.76 C
T_2_	0.68 d	0.69 d	0.69 C	0.22 d	0.21 d	0.22 C	121.52	121.64	121.58 C	6.77 d	6.77 d	6.78 C
T_3_	0.69 d	0.71 d	0.70 C	0.22 d	0.21 d	0.22 C	121.52	122.00	122.76 C	6.77 d	6.80 d	6.79 C
T_4_	0.96 c	1.24 ab	1.10 B	0.32 c	0.38 b	0.35 B	178.85	190.00	184.43 B	8.09 c	9.28 b	8.69 B
T_5_	1.12 b	1.37 a	1.25 A	0.38 b	0.45 a	0.42 A	195.00	206.65	200.83 A	9.25 b	10.31 a	9.78 A
Mean (Y)	0.82 B	0.94 A		0.27 B	0.29 A		147.67	152.45		7.53 B	7.99 A	
*LSD* (*p ≤ 0.05*)	*T* = *0.10*; *Y* = *0.06*; *T* × *Y* = *0.14*			*T* = *0.03*; *Y* = *0.01*; *T* × *Y* = *0.03*			*T* = *8.43*			*T* = *0.41*; *Y* = *0.26*; *T* × *Y* = *0.58*		

Figures of interaction and main effects sharing the same case letter do not differ significantly (*p* ≤ 0.05) by the least significant difference test; likewise, the figures of main effects and interaction without lettering do not differ significantly (*p* ≤ 0.05) by the least significant difference test; T_1_ = Control (plots with no crop residues or extract application); T_2_ = Sorghum water extract at 10 L ha^−1^; T_3_ = Sorghum water extract @ 20 L ha^−1^; T_4_ = Sorghum residues at 4 t ha^−1^; T_5_ = Sorghum residues at 6 t ha^−1^; ^(a)^ T = treatments; ^(b)^ Y = year.

## Data Availability

Not applicable.

## References

[1] Islam S.M.F., Karim Z. (2019). World’s demand for food and water: The consequences of climate change. Desal. Chall. Opport..

[2] World Resources Institute (2014). Creating a Sustainable Food Future. Report 2013–2014: Interim Findings.

[3] Tian X., Engel B.A., Qian H., Hua E., Sun S., Wang Y. (2021). Will reaching the maximum achievable yield potential meet future global food demand?. J. Clean. Prod..

[4] Liu Y., Liu X., Liu Z. (2022). Effects of climate change on paddy expansion and potential adaption strategies for sustainable agriculture development across Northeast China. Appl. Geogr..

[5] Khan M.T., Rafi M.A., Sultana R., Munir A., Ahmad S. (2022). Diversity and Bio-Geography of Subfamily Eumeninae (Vespidae: Hymenoptera) in Sindh, Pakistan. Pak. J. Zool..

[6] dos Reis J.C., Rodrigues G.S., de Barros I., Rodrigues R.D.A.R., Garrett R.D., Valentim J.F., Smukler S. (2021). Integrated crop-livestock systems: A sustainable land-use alternative for food production in the Brazilian Cerrado and Amazon. J. Clean. Prod..

[7] Farooq N., Abbas T., Tanveer A., Jabran K. (2020). Allelopathy for weed management. Coevol. Sec. Metab..

[8] Sharma A., Kumar V., Shahzad B., Tanveer M., Sidhu G.P.S., Handa N., Thukral A.K. (2019). Worldwide pesticide usage and its impacts on ecosystem. SN Appl. Sci..

[9] Nadeem M.A., Abbas T., Tanveer A., Maqbool R., Zohaib A., Shehzad M.A. (2017). Glyphosate hormesis in broad-leaved weeds: A challenge for weed management. Arch. Agron. Soil Sci..

[10] Peerzada A.M., O’Donnell C., Adkins S. (2019). Optimizing Herbicide Use in Herbicide-Tolerant Crops: Challenges, Opportunities, and Recommendations. Agron. Crops.

[11] Farooq M., Jabran K., Cheema Z.A., Wahid A., Siddique K.H.M. (2011). Role of allelopathy in agricultural pest management. Pest Manag. Sci..

[12] Macias F.A. (2002). New approaches in allelopathy, challenge for the new millenium. Third World Congr. Allelopath. Abstr..

[13] Islam A.M., Yeasmin S., Qasem J.R.S., Juraimi A.S., Anwar M.P. (2018). Allelopathy of medicinal plants: Current status and future prospects in weed management. Agric. Sci..

[14] Khaliq A., Matloob A., Hussain A., Hussain S., Aslam F., Zamir S.I., Chattha M.U. (2015). Wheat residue management options affect productivity, weed growth and soil properties in direct-seeded fine aromatic rice. Clean Soil Air Water.

[15] Qi G., Li N., Sun X.S., Wang D. (2019). Overview of sorghum industrial utilization. Sorghum State Art Future Perspect..

[16] Hussain M.I., Danish S., Sánchez-Moreiras A.M., Vicente Ó., Jabran K., Chaudhry U.K., Reigosa M.J. (2021). Unraveling sorghum allelopathy in agriculture: Concepts and implications. Plants.

[17] Farooq M., Khan I., Nawaz A., Cheema M.A., Siddique K.H. (2020). Using sorghum to suppress weeds in autumn planted maize. Crop Prot..

[18] Cheema Z.A., Khaliq A. (2000). Use of sorghum allelopathic properties to control weeds in irrigated wheat in a semi-arid region of Punjab. Agric. Ecosyst. Environ..

[19] Cheema Z.A., Khaliq A., Akhtar S. (2001). Use of sorghum water extract as a natural weed inhibitor in spring mung bean. Int. J. Agric. Biol..

[20] More S.S., Shinde S.E., Kasture M.C. (2020). Root exudates a key factor for soil and plant: An overview. Pharma Innov. J..

[21] Kumar A., Dubey A. (2020). Rhizosphere microbiome: Engineering bacterial competitiveness for enhancing crop production. J. Adv. Res..

[22] Komal N., Zaman Q.U., Yasin G., Nazir S., Ashraf K., Waqas M., Ahmad M., Batool A., Talib I., Chen Y. (2022). Carbon Storage Potential of Agroforestry System near Brick Kilns in Irrigated Agro-Ecosystem. Agriculture.

[23] Bao Y., Dolfing J., Guo Z., Chen R., Wu M., Li Z., Feng Y. (2021). Important ecophysiological roles of non-dominant Actinobacteria in plant residue decomposition, especially in less fertile soils. Microbiome.

[24] Ferdous Z., Zulfiqar F., Datta A., Hasan A.K., Sarker A. (2021). Potential and challenges of organic agriculture in Bangladesh: A review. J. Crop Improv..

[25] Scavo A., Abbate C., Mauromicale G. (2019). Plant allelochemicals: Agronomic, nutritional and ecological relevance in the soil system. Plant Soil..

[26] Ryan J., Estefan G., Rashid A. (2001). Soil and Plant Analysis: Laboratory Manual; International Center for Agricultural Research in Dry Areas (ICARDA): Aleppo, Syria.

[27] Blake G.R., Hartge K.H., Lute A. (1986). Bulk density. Methods of Soil Analysis: Part 1. Physical and Mineroalogical Methods.

[28] Vomocil J.A., Blake C.A. (1965). Porosity. Methods of Soil Analysis.

[29] Bremner J.M., Mulvaney C.S., Page A.L., Miller R.H., Keeny D.R. (1982). Total nitrogen. Methods of Soil Analysis.

[30] Walkley A., Black I.A. (1934). An examination of Degtjareff method for determining soil organic matter and aproposed modification of the chromic acid titration method. Soil Sci..

[31] Olsen S.O., Sommers I.E., Page A.L. (1982). Phosphorus. Methods of Soil Analysis.

[32] Helmke P.A., Sparks D.L. (1996). Lithium, sodium and potassium, rubidium and cesium. Methods of Soil Analysis.

[33] Aslam Z., Yasir M., Jeon C.O., Chung Y.R. (2008). Lysobacter oryzae sp. nov., isolated from the rhizosphere of rice (*Oryza sativa* L.) managed under no-tillage practice. Int. J. Syst. Evol. Microbiol..

[34] Janssen P.H., Yates P.S., Grinton B.E., Taylor P.M., Sait M. (2002). Improved culturability of soil bacteria and isolation in pure culture of novel members of the divisions Acidobacteria, Actinobacteria, Proteobacteria, and Verrucomicrobia. Appl. Environ. Microbiol..

[35] Wu W.X., Ye Q.F., Min H., Duan X.J., Jin W.M. (2004). Bt-transgenic rice straw affects the culturable micro biota and dehydrogenase and phosphatase activities in a flooded paddy soil. Soil Biol. Biochem..

[36] Martin J.P. (1950). Use of acid, rose bengal and streptomycin in the plate method for enumerating soil fungi. Soil Sci..

[37] Min H., Ye Y.F., Chen Z.Y., Wu W.X., Du Y.F. (2001). Effects of butachlor on microbial populations and enzyme activities in paddy soil. J. Environ. Sci. Health.

[38] Tabatabai M.A., Bremner J.M. (1969). Use of p-nitrophenyl phosphate for assay of soil phosphatase activity. Soil Biol. Biochem..

[39] Rab A., Khan M.R., Haq S.U., Zahid S., Asim M., Afridi M.Z., Munsif F. (2016). Impact of biochar on mungbean yield and yield components. Pure Appl. Biol..

[40] Won O.J., Uddin M.R., Park K.W., Pyon J.Y., Park S.U. (2013). Phenolic compounds in sorghum leaf extracts and their effects on weed control. Allelopath. J..

[41] Ullah R., Aslam Z., Maitah M., Zaman Q., Bashir S., Hassan W., Chen Z. (2020). Sustainable weed control and enhancing nutrient use efficiency in crops through Brassica (*Brassica compestris* L.) allelopathy. Sustainability.

[42] Cheema Z.A., Khaliq A., Abbas M., Farooq M. (2007). Allelopathic potential of sorghum (*Sorghum bicolor* L. Moench) cultivars for weed management. Allelopath. J..

[43] Essien B., Essien J., Nwite J., Eke K., Anaele U., Ogbu J. (2009). Effect of organic mulch materials on maize performance and weed growth in the derived savanna of South Eastern Nigeria. Nigeria Agric. J..

[44] Zaji B., Majd A. Allelopathic potential of canola (*Brassica napus* L.) residues on weed suppression and yield response of maize (*Zea mays* L.). Proceedings of the International Conference on Chemical, Ecology and Environmental Sciences (IICCEES).

[45] Kamara A., Akobundu I., Chikoye D., Jutzi S. (2000). Selective control of weeds in an arable crop by mulches from some multipurpose trees in south western Nigeria. Agrofor. Sys..

[46] Khaliq A., Hussain S., Matloob A., Tanveer A., Aslam F. (2014). Swine cress (*Cronopus didymus* L. Sm.) residues inhibit rice emergence and early seedling growth. Phillipine Agric. Sci..

[47] Khaliq A., Matloob A., Farooq M., Mushtaq M.N., Khan M.B. (2011). Effect of crop residues applied isolated or in combination on the germination and seedling growth of horse purslane (*Trianthema portulacastrum*). Planta Daninha.

[48] Khaliq A., Matloob A., Irshad M.S., Tanveer A., Zamir M.S.I. (2010). Organic weed management in maize through integration of allelopathic crop residues. Pak. J. Weed Sci. Res..

[49] Khanh T.D., Chung M.I., Xuan T.D., Tawata S. (2005). The exploitation of crop allelopathy in sustainable agricultural production. J. Agron. Crop Sci..

[50] Jabran K., Mahajan G., Sardana V., Chauhan B.S. (2015). Allelopathy for weed control in agricultural systems. Crop Protect..

[51] Shehzad T., Okuno K. (2020). Genetic analysis of QTLs controlling allelopathic characteristics in sorghum. PLoS ONE.

[52] Alsaadawi I.S., Khaliq A., Lahmod N.R., Matloob A. (2011). Weed management in broad bean (*Vicia faba* L.) through allelopathic *Sorghum bicolor* (L.) Moench residues and reduced rate of a pre plant herbicide. Allelopath. J..

[53] Kruidhof H., Bastiaans L., Kropff M. (2008). Ecological weed management by cover cropping: Effects on weed growth in autumn and weed establishment in spring. Weed Res..

[54] Stagnari F., Galieni A., Speca S., Cafiero G., Pisante M. (2014). Effects of straw mulch on growth and yield of durum wheat during transition to conservation agriculture in Mediterranean environment. Field Crops Res..

[55] Alam M.K., Islam M.M., Salahin N., Hasanuzzaman M. (2014). Effect of tillage practices on soil properties and crop productivity in wheat mungbean rice cropping system under subtropical climatic conditions. Sci. World J..

[56] Adugna A., Abegaz A. (2016). Effects of land use changes on the dynamics of selected soil properties in northeast Wellega, Ethiopia. Soil.

[57] Marchiosi R., dos Santos W.D., Constantin R.P., de Lima R.B., Soares A.R., Finger-Teixeira A., Mota T.R., de Oliveira D.M., Foletto-Felipe M.D.P., Abrahão J. (2020). Biosynthesis and metabolic actions of simple phenolic acids in plants. Phytochem. Rev..

[58] Raza T., Khan M.Y., Nadeem S.M., Imran S., Qureshi K.N., Mushtaq M.N., Eash N.S. (2021). Biological management of selected weeds of wheat through co-application of allelopathic rhizobacteria and sorghum extract. Biol. Control.

[59] Nawaz A., Lal R., Shrestha R.K., Farooq M. (2016). Mulching affects soil properties and greenhouse gases emissions under long term no-till and plough till systems in Alfisol of central Ohio. Land Develop. Degrad..

[60] Vilkiene M., Mockeviciene I., Karcauskiene D., Suproniene S., Doyeni M.O., Ambrazaitiene D. (2021). Biological indicators of soil quality under different tillage systems in retisol. Sustainability.

[61] Duke O.S. (2015). Proving Allelopathy in crop-weed interactions. Weed Sci..

[62] Rathore S.S.S., Shekhawat K. (2022). Crop Residue Recycling for Improving Crop Productivity and Soil Health. Handbook of Research on Green Technologies for Sustainable Management of Agricultural Resources.

[63] Farhangi-Abriz S., Ghassemi-Golezani K., Torabian S. (2021). A short-term study of soil microbial activities and soybean productivity under tillage systems with low soil organic matter. Appl. Soil Ecol..

[64] Głąb L., Sowiński J., Bough R., Dayan F.E. (2017). Allelopathic potential of sorghum (*Sorghum bicolor* (L.) Moench) in weed control: A comprehensive review. Adv. Agron..

[65] Burezq H., Davidson M.K. (2021). Ecological Intensification for Soil Management: Biochar—A Natural Solution for Soil from Agricultural Residues. Sustainable Intensification for Agroecosystem Services and Management.

[66] Ravankar H.N., Patil R., Puranik R.B. (2000). Decomposition of different organic residues in soil. PKV Res. J..

[67] Anda P. (2021). The reciprocal effect between soil water content and the soil bulk density on the growth and yield of onion (*Allium cepa* L.). J. Appl. Agric. Sci. Technol..

[68] Andrews E.M., Kassama S., Smith E.E., Brown P.H., Khalsa S.D.S. (2021). A review of potassium-rich crop residues used as organic matter amendments in tree crop agroecosystems. Agriculture.

[69] Krishna G.A., Misra A.K., Hati K.M., Bandyopadhyay K.K., Ghosh P.K., Mohanty M. (2004). Rice residue management options and effects on soil properties and crop productivity. Food Agric. Environ..

[70] Verhulst N., Nelissen V., Jespers N., Haven H., Sayre K.D., Raes D., Deckers J., Govaerts B. (2011). Soil water content, maize yield and its stability as affected by tillage and crop residue management in rainfed semi-arid highlands. Plant Soil.

[71] Nazir S., Zaman Q.U., Abbasi A., Komal N., Riaz U., Ashraf K., Ahmad N., Agerwal S., Chen Y. (2021). Bioresource Nutrient Recycling in the Rice–Wheat Cropping System: Cornerstone of Organic Agriculture. Plants.

[72] Zhou J., Xu D., Xue C. (2002). Study of comprehensive utilization efficiency of returning rice straw to field. Chin. Agric. Sci. Bull..

[73] Jin Y.Q., Du D.J., Gao H.J., Chang J., Zhang L.G. (2013). Effects of maize straw returning on water dynamics and water use efficiency of winter wheat in lime concretion black soil. J. Triticeae Crops.

[74] Raut V.U., Bhowate R.T., Waghmare A.G. (2010). Effect of crop residues on nutrient contents in green gram-sunflower cropping sequence. Green Farm.

[75] Saqib S., Uddin S., Zaman W., Ullah F., Ayaz A., Asghar M., Rehman S., Munis M.F.H., Chaudhary H.J. (2021). Characterization and phytostimulatory activity of bacteria isolated from tomato (*Lycopersicon esculentum* Mill.) rhizosphere. Microb. Pathog..

[76] Abbas T., Ahmad A., Kamal A., Nawaz M.Y., Jamil M.A., Saeed T., Ateeq M. (2021). Ways to use allelopathic potential for weed management: A review. Int. J. Food Sci. Agric..

[77] Hussain M.I., Reigosa M.J. (2021). Secondary metabolites, ferulic acid and *p*-hydroxybenzoic acid induced toxic effects on photosynthetic process in *Rumex acetosa* L.. Biomolecules.

